# Dry eye disease severity and impact on quality of life in type II diabetes mellitus

**DOI:** 10.3389/fmed.2023.1103400

**Published:** 2023-02-27

**Authors:** Tetiana Zhmud, Natalia Malachkova, Robert Rejdak, Ciro Costagliola, Marina Concilio, Galyna Drozhzhyna, Damiano Toro Mario, Svitlana Veretelnyk

**Affiliations:** ^1^Department of Ophthalmology, National Pirogov Memorial Medical University, Vinnytsia, Ukraine; ^2^Department of General and Pediatric Ophthalmology, Medical University of Lublin, Lublin, Poland; ^3^Eye Clinic, Department of Neurosciences, Reproductive and Odontostomatological Sciences, University of Naples Federico II, Naples, Italy; ^4^Department of Medicine and Health Sciences “V. Tiberio”, University of Molise, Campobasso, Italy; ^5^Department of Corneal Pathology, SI “Filatov Institute of Eye Diseases and Tissue Therapy of the NAMS of Ukraine”, Odesa, Ukraine; ^6^Eye Clinic, Public Health Department, University of Naples Federico II, Naples, Italy

**Keywords:** dry eye disease, quality of life, ocular surface disease index, Oxford scale, type 2 diabetes mellitus

## Abstract

**Aim:**

To assess the severity of dry eye disease (DED) in humans, its impact on quality of life (QoL) and to grade the damage incurred by the anterior ocular surface in patients diagnosed with type 2 diabetes mellitus (T2DM).

**Patients and methods:**

Forty-six patients (mean age ± SD = 63.8 ± 6.7 years) diagnosed with T2DM were enrolled in the experimental group and 26 healthy individuals constituted the control group (67.9 ± 8.9 years). The diagnosis and gradation of DED were conducted in accordance with the International Task Force severity grading scheme. Disease-specific questionnaires were used to obtain the Ocular Surface Disease Index (OSDI) and assess the negative effects of the disease on the patient’s QoL. The severity of conjunctival redness and corneal/conjunctival staining was assessed by Efron and Oxford scales, respectively.

**Results:**

According to OSDI scores, the entire experimental group presented symptoms of DED: 54.4% were diagnosed with mild DED and 46.6% with moderately severe DED. No cases of severe DED were diagnosed in either the experimental or control group. In the control group, 57.7% of individuals did not have the disease. A significant difference between the experimental and control groups was recorded for both OSDI scores (*p*  < 0.01) and health-related QoL (*p* < 0.01). It was observed that keratopathy influenced the mean OSDI values of patients. The mean OSDI value was 25.14 ± 3 in the experimental group diagnosed with keratopathy, 19.3 ± 3.5 in the subgroup with no indications of corneal injury (*p* = 0.000002), and 13.0 ± 3.0 in the control group (*p*  <  0.000002). Based on the DEWS scheme, a grade I severity level was observed in 46% of control subjects and 33% of patients diagnosed with T2DM (*p* = 0.4915); grades II and III were detected in the bulk of the experimental group (*p* = 0.0051; *p* = 0.1707). None of the subjects in the control or experimental groups manifested grade IV severity of DED.

**Conclusion:**

In comparison to healthy adults, DED adversely impacts the QoL of type 2 DM patients, regardless of the disease’s association with keratopathy.

## 1. Introduction

The 10th edition of the International Diabetes Atlas (2021) predicts an increase in the global prevalence of diabetes mellitus (DM) from 537 million in 2021 to 786 million in 2045 (1). Indeed, a steady surge has been observed in the number of patients diagnosed with type 2 diabetes mellitus (T2DM) (2) with about 541 million adults reporting impaired glucose tolerance, a powerful predisposing factor for T2DM (1).

DM can affect all ocular tissues. Patients with DM manifest symptoms in both anterior and posterior segments of the eye, causing ailments such as blepharitis, cataract, diabetic retinopathy, and macular edema. Dry eye has been recognized as a ubiquitous ocular symptom in diabetic patients. Nevertheless, this condition is often overlooked by healthcare providers (3).

Hom et al. reported that 53% of patients with either diabetes or glucose intolerance were diagnosed with clinically relevant dry eye disease (DED) (4). Yu et al. Have also been able to demonstrate a positive correlation between proliferative diabetic retinopathy and tear film dysfunction (5).

The Dry Eye Workshop II of the Tear Film and Ocular Surface Society (TFOS DEWS II) defined DED as “A multifactorial disease of the ocular surface characterized by a loss of homeostasis in the tear film and accompanied by ocular symptoms, in which tear film stability and hyperosmolarity, ocular surface inflammation and damage, and neurosensory abnormalities play etiological roles” (6). About 344 million people worldwide have either been directly or indirectly afflicted by DED, marking it as a growing global medical concern. DED is markedly more prevalent in diabetics than in healthy subjects and also more common in people with T2DM than in type 1 diabetes mellitus (T1DM) (7, 8). Recent publications have reported that the prevalence of DED ranges from 36 to 72% in T2DM patients (9–11). Pathophysiologically, DED is a multifactorial disorder; several intrinsic and extrinsic factors may further worsen this condition, such as diabetes, immunological and metabolic disturbances. Chronic hyperglycemia results in abnormal tear dynamics and osmolarity, activation of the inflammatory cascade, and innate immune responses which, in turn, induce oxidative stress (12). Persistent symptoms of DED, such as visual disturbance, blurred vision, ocular discomfort, burning, foreign body sensation, and photophobia affect the physical as well as the mental quality of life (QoL), reducing both to below-healthy standards (13, 14). Patient responses in questionnaires on QoL are important tools to evaluate and document psychometric characteristics of well-being and levels of independence (15) of such a drastically expanding population. Several psychometric tests have been developed and validated for the assessment of health and QoL in patients with DED. Specifically, the Ocular Surface Disease Index (OSDI) score has been designed to expeditiously assess ocular symptoms consistent with DED, their impact on vision, and eventually on QoL. To our knowledge, this is the first study assessing the inclemency of DED, its prevalence, and its impact on QoL. We have also aimed to grade the damage of the anterior ocular surface in patients with T2DM, based on DEWS, compared to a healthy individual.

## 2. Materials and methods

### 2.1. Study design

This study was designed to be prospective and observational and performed at Vinnytsia Regional Clinical Hospital, named after “N.I. Pirogov,” from June to December 2021. The study protocol was approved by the local Committee of Bioethics of the National Pirogov Memorial University. Procedures were performed following the Declaration of Helsinki. Written and informed consent was obtained from all patients included in the study.

All consecutive patients addressed to the eye clinic with a diagnosis of T2DM were enrolled. Healthy individuals, sex- and age-matched, were constituted as the control group.

Exclusion criteria comprised of prior history in ocular surgery, use of ocular topical treatment within seven days of the study, and affliction with systemic diseases considered as independent risk factors for DED (Sjogren syndrome, rheumatoid arthritis, systemic lupus erythematosus, ankylosing spondylitis). The majority of T2DM patients included in the study had standard glycemic control (mean HbA1c = 7.0 ± 0.7%, range from 5.6 to 9.0%).

All the patients underwent a thorough ophthalmologic examination and specific tests to evaluate tear film quantity (Schirmer test) and quality (fluorescein tear break-up time (TBUT)).

The International Task Force severity grading scheme (dry eye severity grading scheme), recommended by DEWS, was used to diagnose and grade DED (2007) (16, 17). This scheme is based on nine parameters and classifies DED into four severity levels. The nine parameters include self-reported discomfort, palpable signs, and symptoms noted in ocular surface examination (conjunctival redness and staining, corneal staining, corneal/tear signs, meibomian gland dysfunction (MGD), TBUT, Schirmer test). The Efron grading scale was used to evaluate the degree of conjunctival redness (18), while the Oxford Scheme was used for corneal/conjunctival staining (19).

The Oxford Scheme is a grading scale consisting of a panel series marked A to E, representing patterns, used as standard images to grade the degree of staining observed in patients with DED. Severity is assessed by the number of punctate dots recorded on slip-lamp examination (19).

Quality-of-life (QoL), appertaining to health, was gauged by the OSDI (Allergan, Irvine, CA, United States) as per the recommendation of the TFOS DEWS II (6). The responses of patients to custom questionnaires were reliable and valid for both DED and QoL assessment owing to satisfactory psychometric elements (20). The questionnaire rating consisted of twelve modules distributed into three subscales, *viz.*, ocular symptoms, vision functionalities, and environmental triggers. For each module, patients asserted a particular frequency and/or severity of the symptom on the five-point Likert scale. The all-inclusive score had a range of 0 to 100, with a cut-off value of 12 (positive for DED if the score is ≥13). DED could then be classified as mild (from 13 to 22), moderate (23–32), and severe (≥ 33) based on the scores obtained (21).

### 2.2. Statistics

The software suite STATISTICA v.10.0 (StatSoft, Europe) was used for data analysis in this study. Continuous variables were analyzed as mean value ± standard deviation (SD), while absolute variables were measured in terms of proportions. An independent 2-tailed *t*-test was conducted for comparing quantitative variables with the bell curve (OSDI, presence of keratopathy), whereas Fisher’s exact test was used for the comparison of qualitative variables. The relationship between OSDI and the severity of DED was evaluated using Spearman’s correlation analysis. *p*-values <0.05 were considered statistically significant.

## 3. Results

Forty-six patients diagnosed with T2DM and DED were included in the experimental group (mean age ± SD = 63.8 ± 6.7 years) and twenty-six age- and sex-matched healthy participants were enrolled in the control group (mean age ± SD =67.9 ± 8.9 years). Table 1 displays the demographic as well as the clinical data of participants from both experimental and control groups. The acuteness of DED was assessed through the OSDI questionnaire (Table 2) and the severity scheme issued by DEWS. The OSDI score ascribed DED symptoms to all the patients of the experimental group. Twenty-five patients, representing 54.4% of the experimental group, and 11 patients comprising 42.3% of the control group were diagnosed with mild DED and moderate DED was reported only by 21 patients (45.6%) in the experimental group. No patients with severe DED were identified in either the experimental or the control group. QoL apropos health differed significantly in the experimental and control groups (*p* < 0.01). Patients in the experimental group were further divided into two subgroups based on the presence and absence of corneal involvement. By comparing the mean values of the OSDI scores, we were able to record significant variations between the three groups, as can be inferred from Table 3. The mean OSDI score was 25.14 ± 3 for the experimental subgroup diagnosed with keratopathy and 19.3 ± 3.5for the subgroup with no corneal injury (*p* = 0.000002). Additionally, 32 of the 46 patients (69.6%) in the experimental group had a substantially distinct mean OSDI score as compared to controls, suggesting lower QoL in T2DM patients. Retired patients diagnosed with DED experienced eye soreness (*r*_s_ = 0.345, *p* = 0.0188; *r*_s_ = 0. 631, *p* < 0.01) and limited routine activities, mostly watching television and reading (26/58, 44.8% and 24/58, 41.4%, respectively).

**Table 1:** Demographic and clinical characteristics of study and control groups.

		Study group (*n* = 46)	Controls (*n* = 26)	*p*
Age (mean ± SD, years)		63.8 ± 6.7	67.9 ± 8.9	0.043*
Sex(abs.)	Males	19	12	0.805**
	females	27	14	0.805**
Median duration of T2DM(years)		9	N/A	N/A
Mean HbA1c (%)		7.07 ± 0.75	N/A	N/A
Presence of keratopathy(abs.)		14	0	0.001**
Schirmer test (mean ± SD, mm)		8.3 ± 3.56	10.12 ± 2.5	0.024*
TBUT (mean ± SD, seconds)		8.39 ± 2.45	11.15 ± 2	0.000006*
MGD (abs.)		36	20	1.000**

SD, standard deviation; abs, absolute value; T2DM, type 2 diabetes mellitus; Hb1ac, glycated hemoglobin; MGD, Meibomian gland dysfunction; *p*, *p*-value. ^*^Student’s *t*-test, independent, by variables. **Fisher’s exact test, 2-tailed.

**Table 2:** OSDI mean scores and DED grades in the study and control groups.

OSDI	Mean ± SD	Normal	%	Mild 13–22	%	Moderate 23–32	%	Severe 33–100	%
Study group	20.1 ± 4.03	0	0	25	54.4	21	45.6	0	0
Control group	13 ± 3	15	57.7	11	42.3	0	0	0	0
*p*	0.00000*	0.0000**		0.4621		0.0000**		N/A	

OSDI, ocular surface disease index; SD, standard deviation; *p*, *p*-value. *p < 0.01, *t*-value = 7.82, a significant difference between the study and control groups. **p < 0.01, 2-tailed Fisher’s exact test, a significant difference between the study and control groups.

**Table 3:** Impact of keratopathy on OSDI in study and control groups.

Subgroup	OSDI	Mean ± SD	*t*-value	*p*
1	Study group, with keratopathy (*n* = 14)	25.14 ± 3	12.14	0.000000*
2	Study group, without keratopathy (*n* = 32)	19.3 ± 3.5	5.41	0.000002**
3	Control group, without keratopathy (*n* = 26)	13 ± 3	7.26	0.000000***

*A significant difference between subgroups 1 and 3. **Significant difference between subgroups 1 and 2. ***Significant difference between subgroups 2 and 3.

Following regulations in the DEWS scheme, eleven patients of the control group tested negative for DED. Grade I severity level was recorded in 46% of control individuals and 33% of patients with T2DM. Grades II and III were predominantly detected in patients of the experimental group. None of the participants in either control or experimental groups were diagnosed with grade IV DED severity (Table 4). Furthermore, we have been able to accomplish a positive correlation between the mean OSDI score and DEWS grade (*r* = 0.705; *p* < 0.01) in the experimental group and a negative correlation with the Schirmer test and TBUT (Table 5).

**Table 4:** DEWS grades distribution among study and control groups.

DEWS	Normal (*n*)	%	Grade I (*n*)	%	Grade II (*n*)	%	Grade III (*n*)	%	Grade IV (*n*)	%
Study group	0	0	15	33	25	54	6	13	0	0
Control group	11	42	12	46	2	12	0	0	0	0
*p*	0.0001*		0.4915		0.0051*		0.1707		N/A	

*p < 0.01, a significant difference between the study and control groups; 2-tailed, Fisher’s exact test.

**Table 5:** Correlations between OSDI and objective tests in the study group.

Test title	Study group (*n* = 46) (mean ± SD)	*r*_s_	*p*-value
OSDI	20.1 ± 4.03	N/A	N/A
DEWS	1.8 ± 0.65	0.705	0.000000*
Shirmer’s test	8.3 ± 3.56	−0.316	0.032534**
TBUT	8.39 ± 2.45	−0.292	0.048992***

*r*_s_, Spearman’s rank correlation coefficient. *p < 0. 01, a significant difference between OSDI and DEWS. **p < 0.05, a significant difference between OSDI and Shirmer’s test. ***p < 0.05, a significant difference between OSDI and TBUT.

**Figure 1:**
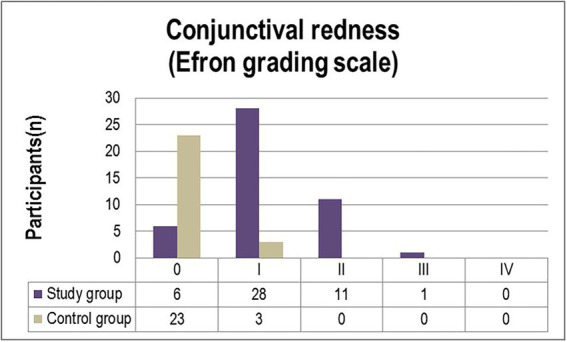
Distribution of severity grades of corneal/conjunctival staining (Oxford scheme) in the study and control groups.

**Figure 2:**
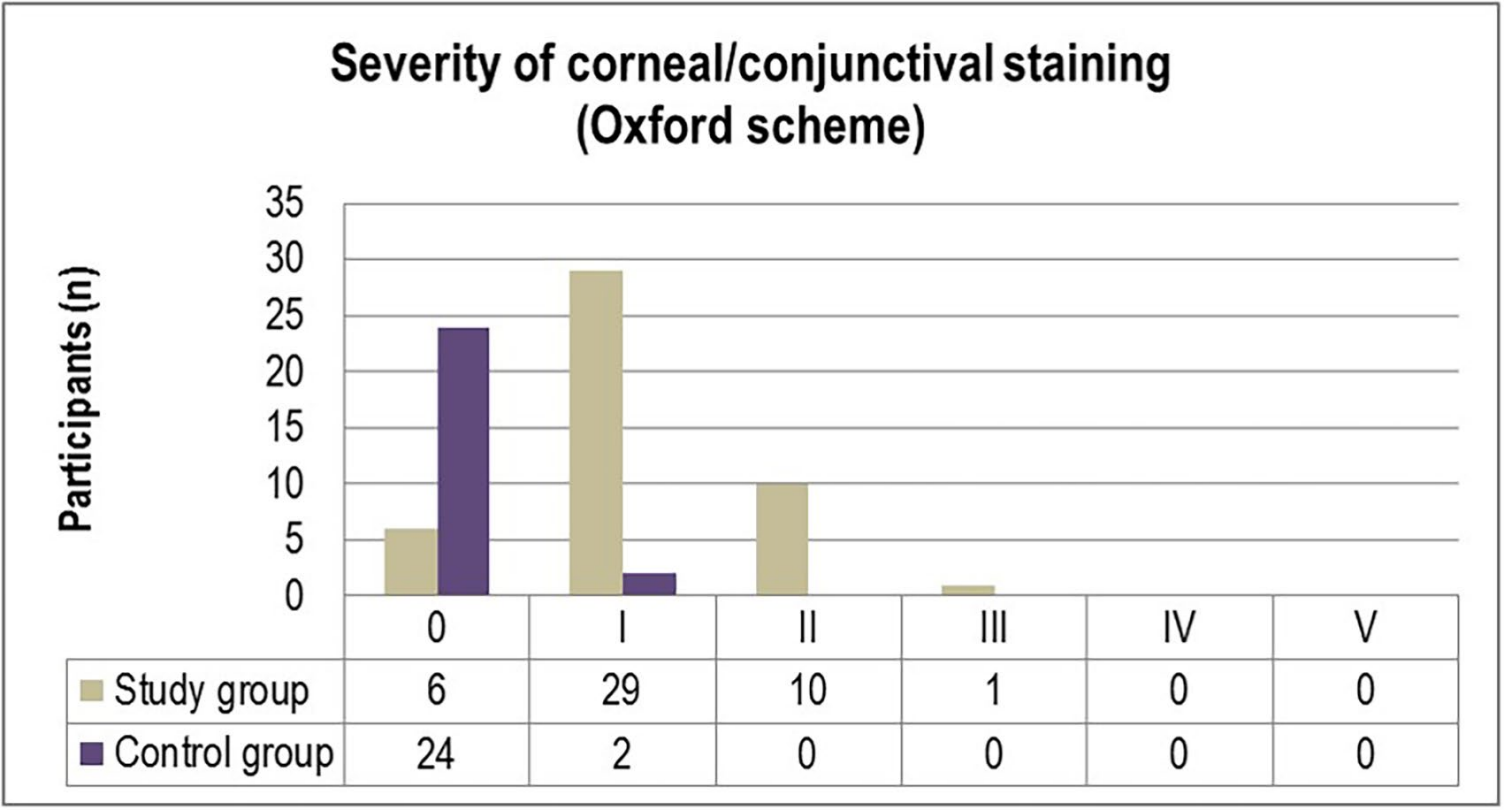
Efron grades distribution in the study and control groups.

The corneal and conjunctival staining, graded using the Oxford scale (Figure 1) ascertained grade I punctate staining in the majority of diabetic patients included in the study (63%). While 21.7% of participants were diagnosed with grade II punctate staining, only one patient (2.2%) displayed grade III fluorescein-stained corneal erosions. In comparison, 7.7% of individuals from the control group were diagnosed with grade I abnormal corneal/conjunctival staining (Figure 1). Pathological patterns associated with conjunctival redness were absent in 88.5% of the control group and 13% of the experimental group (*p* = 0.0001). Twenty-eight T2DM patients (61%) and three control individuals (11.5%) were diagnosed with grade I severity (mild redness of bulbar conjunctiva, slightly engorged major vessels) according to the Efron grading scale (*p* = 0.0078). A majority of subjects from both the experimental and control groups complained of mild discomfort on exposure to environmental triggers. Thorough scrutiny revealed conjunctival and limbal redness with a mild ciliary flush in diabetic patients diagnosed with grade II severity on the Efron scale. One patient from the study group was diagnosed with grade III severity of conjunctival redness associated with keratopathy and a decreased meniscus (Figure 2).

## 4. Discussion

Recently, an extensive demographic study employed the Short Form Health Survey (SF36) to demonstrate a significant reduction in QoL, particularly mental health, of patients diagnosed with DED. Co-morbidities may be minimized, but complete eradication of negative effects has not been demonstrated to date. (14).

All patients recruited in the study group were afflicted with DED with the severity of the disease varying from mild to moderate (54.4 and 45.6, respectively) according to the OSDI scores (mean score ± SD = 20.1 ± 4.03 points). Only 42% of healthy individuals from the control group reported mild DED symptoms (mean score = 13.3). Our data and results differ from those reported by Fuerst et al. in that they noted DED in only 52% of diabetic persons in their study (*n* = 26) with severity ranging from mild to severe (mild 16%, moderate 18%, severe 18%) (22), and from the results of a prospective observational study conducted on 58 diabetic patients by Ribeiro et al. who reported a diagnosis of moderate to severe DED in only 26.2% of the patients (23). Yazdani-Ibn-Taz et al. evaluated QoL based on OSDI in both T2DM-diagnosed patients and T1DM-diagnosed patients (mean score = 33.23 and 26.16, respectively). They noted symptoms indicative of DED in 46.7% (14) of T2DM-diagnosed patients not afflicted with diabetic retinopathy (8). As reported in a recent study by Naik et al. (24), there is a significant positive correlation between poor glycemic control (with abnormal levels of Hb1Ac) and a higher degree of dry eyes. Since all the patients enrolled in our experimental group possessed relatively good glycemic control (HbA1c value ± SD = 7.0 ± 0 0.7%), we can presume a minimal impact on the risk of DED in our patients. Hence, we are inclined to presume that the differences in our findings and previous studies might be caused by poor glycemic control of the patients in the previous studies. Furthermore, we propose diabetic neuropathy as a potential factor that negatively impacts corneal sensitivity and might lead to lower OSDI scores in patients diagnosed with chronic T2DM (median = 9 years, range 1–27 years).

The worldwide prevalence of DED is in the expected range of 5 to 50%, and it is notably higher among subjects over 60 years of age with increasing incidences among the elderly (25, 26). Garcia-Alfaro et al. reported an 80.5% prevalence of DED (mean OSDI =29.20 ± 19.4) among postmenopausal women (54.18 ± 6.84 years) with 37.7% of participants severely diseased, resulting in lower QoL (26). All female participants in our study in both experimental and control groups experienced only mild or moderate DED and no symptoms of severe affliction.

The most common risk factors of diabetic kerato-epitheliopathy include abnormalities of tear dynamics, decreased corneal sensitivity, and impaired regeneration of corneal epithelial cells (27). Fourteen patients in our experimental group (30.4%) were diagnosed with superficial keratopathy. We found that the OSDI score was significantly different between diabetic patients without keratopathy (mean = 19.3 ± 3.5) and healthy controls (mean = 13.3 ± 3.0). We propose that this difference is potentially determined through other QoL-decreasing factors in T2DM patients rather than through damage to the corneal epithelial cells alone. However, we cannot disregard possible bias in the responses of patients to the questionnaires.

Evaluation of the severity of DED through the dry eye severity scheme yielded a positive association between the value scale and OSDI score in the experimental group. The OSDI questionnaire contains both symptoms and signs to assess and compare the patient’s complaints with objective findings. As suggested by Naik et al., the OSDI questionnaire should be an integral part of the ophthalmological examination of diabetic patients to screen all patients for ocular surface changes, especially for patients with a long history of DM and poor glycemic control (24).

No patients presented signs correlated to grade IV severity of DED; neither disabling discomfort nor corneal ulcerations were reported, the Schirmer test ranged from 2 to 15 mm, and minimal TBUT was 3 s. Though 56% of participants from the control group also exhibited DED, based on DEWS, they only had mild disease.

This study has several limitations. For one, the bias in DED severity owing to good glycemic control of patients included in the experimental group. Secondly, the relatively small sample of patients and the unbalanced distribution of patients’ sex in favor of women (59% of the study group population, as DED commonly affects women more than men). The third limitation was the sporadic cases of grade III corneal damage based on the Oxford scheme and on the Efron scale, which impeded statistical analysis of the association between QoL and this subgroup. Therefore, further prospective studies with larger sample sizes might be designed to evaluate how OSDI may vary in patients with T2DB according to the severity of DM. Also, novel diagnostic tests to analyze the complex ocular surface system should be considered in topical therapy. To support our findings, we have nevertheless conducted a thorough analysis of all the subjects in the experimental as well as control groups based on clinical tests and standardized grading scales, as well as an efficient statistical evaluation of all the data collected. To conclude, we demonstrated that DED is associated with lower QoL among patients with type 2 DM, both with and without keratopathy, in comparison to healthy controls.

## 5. Conclusion

Diabetic retinopathy is a commonly occurring, well-documented ocular morbidity. Developing comorbidities, poor glycemic control, advanced age, and even the female sex factor in the advancement of DED in patients diagnosed with DM. This study, in particular, was conducted to assess the effects of DED on the QoL of patients pre-diagnosed with DM. This study has been able to ascertain that mean OSDI values are commensurate with the DEWS grades used to determine the severity of DED in patients of the experimental group. Through expeditious data collection and methodical statistical analysis, we have been able to determine a substantial positive correlation between lower OSDI scores and corneal sensitivity caused by diabetic neuropathy. This study has also determined that DED negatively impacts T2DM patients, irrespective of their association with keratopathy. The OSDI scores employed to evaluate the ocular surfaces in subjects of both the control and the experimental groups were also salutary in determining the adverse effects of DED on patients’ QoL. A comparison of health-associated QoL of healthy individuals with DED-diagnosed patients offers insights into the challenges faced by patients in performing daily routine tasks. Early detection and subsequent intervention will be pragmatic in dealing with DED. Hence, as has already been suggested, OSDI questionnaires must be essentially integrated into the ophthalmic examination of diabetic patients.

## Data availability statement

The raw data supporting the conclusions of this article will be made available by the authors, without undue reservation.

## Ethics statement

The study was reviewed and approved by local Committee of Bioethics of National Pirogov Memorial University. The patients/participants provided their written informed consent to participate in this study.

## Author contributions

TZ: conceptualization and writing. TZ and SV: formal, statistical analysis, and investigation. NM: data curation. GD: methodology. CM: review and editing. TM: review. TM, RR, and CC: validation. TZ, NM, and GD: original draft. All authors contributed to the article and approved the submitted version.

## Conflict of interest

The authors declare that the research was conducted in the absence of any commercial or financial relationships that could be construed as a potential conflict of interest.

## Publisher’s note

All claims expressed in this article are solely those of the authors and do not necessarily represent those of their affiliated organizations, or those of the publisher, the editors and the reviewers. Any product that may be evaluated in this article, or claim that may be made by its manufacturer, is not guaranteed or endorsed by the publisher.
