# Corrigendum: Dry eye disease severity and impact on quality of life in type II diabetes mellitus

**DOI:** 10.3389/fmed.2023.1218260

**Published:** 2023-05-30

**Authors:** Tetiana Zhmud, Natalia Malachkova, Robert Rejdak, Ciro Costagliola, Marina Concilio, Galyna Drozhzhyna, Damiano Toro Mario, Svitlana Veretelnyk

**Affiliations:** ^1^Department of Ophthalmology, National Pirogov Memorial Medical University, Vinnytsia, Ukraine; ^2^Department of General and Pediatric Ophthalmology, Medical University of Lublin, Lublin, Poland; ^3^Eye Clinic, Department of Neurosciences, Reproductive and Odontostomatological Sciences, University of Naples Federico II, Naples, Italy; ^4^Department of Medicine and Health Sciences “V. Tiberio”, University of Molise, Campobasso, Italy; ^5^Department of Corneal Pathology, SI “Filatov Institute of Eye Diseases and Tissue Therapy of the NAMS of Ukraine”, Odesa, Ukraine; ^6^Eye Clinic, Public Health Department, University of Naples Federico II, Naples, Italy

**Keywords:** dry eye disease, quality of life, ocular surface disease index, Oxford scale, type 2 diabetes mellitus

In the published article, an author name was incorrectly written as Robert Redjak. The correct spelling is Robert Rejdak.

The authors apologize for this error and state that this does not change the scientific conclusions of the article in any way. The original article has been updated.

